# Effects of Compound Polysaccharides Derived from *Astragalus* and *Glycyrrhiza* on Growth Performance, Meat Quality and Antioxidant Function of Broilers Based on Serum Metabolomics and Cecal Microbiota

**DOI:** 10.3390/antiox11101872

**Published:** 2022-09-21

**Authors:** Yingying Qiao, Yongpeng Guo, Wei Zhang, Weibing Guo, Kyselov Oleksandr, Nataliia Bozhko, Zhixiang Wang, Changzhong Liu

**Affiliations:** 1 College of Animal Science and Technology, Henan Agricultural University, Zhengzhou 450046, China; 2 Department of Biochemistry and Biotechnology, Sumy National Agrarian University, 40000 Sumy, Ukraine; 3 Henan Institute of Science and Technology, College of Animal Science, Xinxiang 453003, China; 4 Inner Mongolia Evergrand Pharmaceutical Co., Ltd., Chifeng 025250, China; 5 Department of Biophysics, Biochemistry, Pharmacology and Biomolecular Engineering, Medical Institute, Sumy State University, 40018 Sumy, Ukraine

**Keywords:** *Astragalus*, *Glycyrrhiza*, polysaccharides, broiler, meat quality, antioxidant function, cecal microbiota, metabolomics

## Abstract

This study aimed to evaluate the effects of dietary supplementation of compound polysaccharides derived from *Astragalus* and *Glycyrrhiza* on growth performance, meat quality, antioxidant function, cecal microbiota and serum metabolomics of broilers. A total of 480 one-day-old male Arbor Acres (AA) broilers were randomly divided into four treatments with six replicates comprising 20 broilers each. Treatments: CON group was the basal diet; ANT group was supplemented with Terramycin calcium; LAG group was supplemented with 150 mg/kg *Astragalus* polysaccharides and 75 mg/kg *Glycyrrhiza* polysaccharides; HAG group was supplemented with 300 mg/kg *Astragalus* polysaccharides and 150 mg/kg *Glycyrrhiza* polysaccharides. The results showed that LAG and HAG supplementation increased growth performance, antioxidant function and meat quality compared with the CON group and ANT group and, especially, the effect of LAG treatment was better than HAG. Analysis of cecal microbiota showed that LAG and HAG supplementation altered cecal microbial diversity and composition in broilers. Serum metabolomics analysis showed that a total of 193 differential metabolites were identified in CON and LAG groups, which were mainly enriched in linoleic acid metabolism and glutathione metabolism pathways. Moreover, there was a close correlation between serum metabolites, cecal microbiota and phenotypic indicators. Conclusion: Dietary supplementation of 150 mg/kg Astragalus polysaccharides and 75 mg/kg Glycyrrhiza polysaccharides could improve the growth performance, antioxidant function and meat quality of broilers by changing the serum metabolites and cecal microbiota composition.

## 1. Introduction

The unreasonable use of antibiotics can easily lead to bacterial resistance, resulting in drug residues in meat, eggs and milk, causing food safety problems [1,2] and harming human health and environmental safety [3]. Considering food safety and human health, some countries, such as those of the European Union, the United States, China and India, have promulgated regulations prohibiting or restricting antibiotic growth promoters [4]. Thus, it is urgent to develop green feed additives to replace antibiotics. As feed additives, plant polysaccharides have safety characteristics, no resistance and minor toxic and side effects. Rational addition to the feed can improve the production performance, intestinal microecology, antioxidant function and livestock and poultry product quality [5,6]. Previous studies have shown that polysaccharides extracted from external pistachio hull could partly prevent enzymatic browning in potato slices [7], inhibit lipid peroxidation, have cytoprotective properties [8], thereby reducing lipid oxidation during refrigeration. and improve meat color stability [9]. Interestingly, supplementing green tea polysaccharides in broilers can also improve pH and a* value of chicken breast muscle, decrease the L* value and b* value, increase the abundance of Bacteroides and Lactobacillus and decrease the abundance of Proteobacteria [10]. Moreover, it has been reported that adding ginseng polysaccharides to the diet could display antioxidant, immunomodulatory properties and anti-tumor functions [11].

The primary component of the water-soluble heteropolysaccharide derived from the dried roots or stems of the traditional Chinese medicine herb *Astragalus* is *Astragalus* polysaccharides (APS), which has immunomodulatory, anti-inflammatory, antioxidant, antibacterial and antiviral properties [12]. Dietary supplementation with APS has been shown to boost juvenile broiler body weight gain, perhaps because of increased digestive enzyme activity and antioxidant ability [13]. Moreover, dietary supplementation of gamma-irradiated *Astragalus* polysaccharides in poultry diets can increase ADFI, reduce FCR [14] and alleviate growth decrease under cyclophosphamide and lipopolysaccharide stress in broilers [15,16]. In mice, APS effectively alleviated high-fat diet-induced metabolic disorders, altering gut microbiota composition and function [17]. *Glycyrrhiza* polysaccharides (GPS) are one of the primary active components of *Glycyrrhiza* and have antioxidant, antibacterial, antiviral, anticancer, anti-inflammatory, immunomodulatory, hypoglycemic, reconciling medicine and other biological activities [18]. Adding 1500 mg/kg *Glycyrrhiza* polysaccharide could increase the activity of serum T-SOD in broilers and inhibit the decrease of T-SOD activity and the increase of MDA content induced by lipopolysaccharide [19]. GPS has the potential to improve broiler growth performance by improving intestinal health and modulating gut microbiota [20]. The compatibility of *Astragalus* and *Glycyrrhiza* can promote the synergistic effect and improve efficacy [21]. However, there are few reports on broilers’ compatible use of *Astragalus* polysaccharides and *Glycyrrhiza* polysaccharides. The present study was carried out to investigate the effects of dietary supplementation of *Astragalus*-*Glycyrrhiza* polysaccharides on the production performance, nutrient apparent metabolic rate, meat quality and antioxidant function of broilers. Moreover, serum non-targeted metabolomics and gut microbiota were analyzed to provide insight into the beneficial effects of dietary *Astragalus-Glycyrrhiza* polysaccharides in broilers. The findings in this study will promote the application of *Astragalus-Glycyrrhiza* polysaccharides as antibiotic alternatives in poultry production.

## 2. Materials and Methods

### 2.1. Astragalus Polysaccharides and Glycyrrhiza Polysaccharides

Inner Mongolia Evergrand Pharmaceutical Co., Ltd. (Inner Mongolia, Tongliao, China) provided APS and GPS. The content of polysaccharides in APS and GPS is 70.23% and 61.36%, respectively.

### 2.2. Experimental Design and Animal Management

A total of 480 one-day-old male Arbor Acres (AA) broilers were randomly divided into four treatments with six replicates comprising 20 broilers each. Treatments: CON group was the basal diet; ANT group was supplemented with Terramycin calcium; LAG group was supplemented with 150 mg/kg *Astragalus* polysaccharides and 75 mg/kg *Glycyrrhiza* polysaccharides; HAG group was supplemented with 300 mg/kg *Astragalus* polysaccharides and 150 mg/kg *Glycyrrhiza* polysaccharides. The corn-soybean meal diet was fed to broilers in the experiment and the formula was divided into two stages, i.e., d1–d21 and d22–d42. The ingredients and nutrient levels of basal diets were formulated to meet the NRC (1994) nutrient requirements of broiler chickens (Supplementary Table S1). The broilers were vaccinated with the Newcastle disease vaccine and the infectious bursal vaccine on days 7 and 14 of the experiment. All broilers had free access to feed and clean water during the experiment. The temperature of the chicken coop was maintained at 33 °C at the age of 1 to 4 d and then reduced by 2 °C per week to a final temperature of around 24 °C.

### 2.3. Growth Performance

On the 21st and 42nd days of the experiment, broilers fasted with free drinking water at 8:00 p.m. Broilers were weighed in duplicate units at 8:00 a.m. on the 22nd and 43rd days and the body weight and feed consumption of the broilers were accurately recorded. The average daily gain (ADG), average daily feed intake (ADFI) and feed conversion ratio (FCR) were calculated.

### 2.4. Serum and Breast Muscle Antioxidant Function

Each duplicate randomly selected one male broiler at 42 d. Blood samples were drawn from the wing vein and immediately transferred to coagulation tubes. After centrifugation (3000× *g* at 4 °C for 15 min), serum samples were collected and kept at −20 °C for analysis. The ipsilateral breast muscle was harvested after the broilers were euthanized. The activities of T-AOC, SOD, GSH-Px and MDA content in serum and breast muscles were determined using kits from Nanjing Jiancheng Bioengineering Institute (Nanjing, China).

### 2.5. Meat Quality

Ipsilateral breast muscle samples were collected and the following meat quality indicators were measured.

**pH:** pH was measured using the Testo 205 pH meter (Testo AG, Lenzkirch, Germany), which was inserted directly into the breast muscle within 45 min and 24 h after the euthanasia of broilers. pH meters were calibrated with standard buffers of pH 4.01 and pH 6.86 before use.

**Color:** About 45 min after the euthanasia of broilers, a colorimeter (CH-400, Konica Minolta Holdings, Inc., Tokyo, Japan) was used and three pieces of breast muscle samples (5 cm × 5 cm × 0.5 cm) were cut vertically. The samples’ L*, a* and b* values were measured thrice and the average value was taken as the final color value.

**Drip loss:** About 2 g of breast muscle (W1) from each broiler was weighed and placed in a sealed plastic bag. The plastic bag was inflated to prevent muscle mass from sticking to the wall and hung in a 4 °C refrigerator for 24 h. The filter paper was used to wipe off the water on the muscle surface. The resulting breast muscle (W2) was weighed. Drip loss was calculated using the equation:

Drip loss = (W1 − W2)/W1 × 100%(1)

**Shearing force:** The breast muscle sample was packed in a plastic bag and placed in a constant-temperature water bath at 80 °C for heating. When the central meat temperature reached 70 °C, the meat was collected and cooled to room temperature. Then, the meat was trimmed into strips with length, width and height of 3, 1 and 1 cm, respectively, along the direction of muscle fibers and cut perpendicular to the direction of muscle fibers by using a digital meat tenderizer (model C-LM3B, Northeast Agricultural University).

**Fatty acid:** The fatty acid content of breast muscle was quantitatively determined by the gas chromatographic method (GB/5009.168-2016) [22]. Results of fatty acids were expressed as the percentage of the total fatty acids identified.

### 2.6. qRT-PCR of Intestinal Antioxidant Enzyme Genes

The expression levels of *SOD1, SOD2* and *GSH-Px* mRNA in the intestinal mucosa of broilers (duodenum, jejunum and ileum) were detected by qRT-PCR. The extraction of total RNA from the samples was performed according to the instructions of the Trizol kit. The concentration of total RNA (OD260/280 = 1.8–2.0) was determined using a microspectrophotometer. The extracted RNA was transcribed into cDNA using the Takara reverse transcription kit. Fluorescence quantitative PCR detection was performed using the Takara fluorescence quantitative kit and the reaction volume was 20 μL. Primers were synthesized by Shanghai Sangon Biological Co., Ltd. (Shanghai, China). The specific information on primers is shown in Supplementary Table S2. GAPDH gene was used as an internal reference and the relative expression of the target gene was calculated using the 2^−ΔΔCT^ method.

### 2.7. Cecal Microbiota

Using the QIAamp DNA Stool Mini Kit (QIAGEN, CA, Hamburg, Germany) by the manufacturer’s instructions, microbial DNA was extracted from the contents of the cecum. With primer pairs 338F (5′-ACTCCTACGGGAGGCACAG-3′) and 806R (5′-GGACTACHVGGGTWTCTAAT-3′), the V3-V4 region of the 16S rRNA gene was amplified. By Majorbio Bio-Pharm Technology Co., Ltd. (Shanghai, China), the 16S rRNA genes were sequenced using the Illumina MiSeq platform (Shanghai, China). The practical readings were obtained by demultiplexing the raw reads, quality-filtering them using Trimmomatic, then merging them using FLASH. Using UPARSE (version 7.11, http://www.drive5.com/uparse/ accessed on 10 July 2021), the acquired high-quality reads were assigned to operational taxonomic units (OTUs) with a 97% similarity and chimera sequences were eliminated by comparison with the Silva database using the UCHIME algorithm. The RDP Classifier examined the taxonomy of the representative sequences for each OTU. The bacterial communities’ diversity, composition and differences were examined on the I-Sanger Cloud Platform, which was made available by the Majorbio Bio-Pharm Technology Co., Ltd. (Shanghai, China).

### 2.8. Metabolite Extraction and Analysis

The 400 µL methanol: water (4:1, *v*/*v*) solution was used to extract the metabolites from 100 µL of accurately weighed serum samples. The mixture was treated with a High throughput Tissue Crusher Wonbio-96c (Shanghai Wan Bo Biotechnology Co., Ltd., Shanghai, China) at 50 Hz for 6 min after being allowed to settle at −20 °C. It was followed by vortexing for 30 s and ultrasonic treatment at 40 kHz for 30 min at 5 °C. The samples were placed at −20 °C for 30 min to precipitate proteins. After centrifugation at 13,000× *g* at 4 °C for 15 min, the supernatant was carefully transferred to sample vials for LC-MS/MS analysis.

The mass spectrometric data were collected using a Thermo UHPLC-Q Exactive Mass Spectrometer equipped with an electrospray ionization (ESI) source operating in either positive or negative ion mode. Peak detection and alignment of raw data were performed in Progenesis QI 2.3 (Nonlinear Dynamics, Waters, Milfod, MA, USA). The final dataset was imported into the SIMCA16.0.2 software package (Sartorius Stedim Data Analytics AB, Umea, Sweden) for multivariate analysis. Orthogonal Partial Least Squares Discriminate Analysis (OPLS-DA) were performed using ropes (Version 1.6.2, http://bioconductor.org/packages/release/bioc/html/ropls.html, accessed on 25 July 2021) R package on Majorbio Cloud Platform (https://cloud.majorbio.com, accessed on 25 July 2021). Variable importance in the projection (VIP) was calculated in the OPLS-DA model. Differential metabolites were identified according to the standard of VIP > 1 and *p* < 0.05. Kyoto Encyclopedia of Genes and Genomes (KEGG) database (http://www.genome.jp/kegg/, accessed on 25 July 2021) was used for pathway enrichment analysis. The correlational heatmaps were generated according to the result of the Pearson correlation analysis. The process was conducted in the environment of a Python package named Scipy Stats (https://docs.scipy.org/doc/scipy/, accessed on 27 July 2021).

### 2.9. Statistical Analysis

Statistical analyses were performed using the SPSS Statistics Software (version 18.0, New York, NY, USA). One-way ANOVA evaluated data and the comparative analysis was conducted using Duncan’s test. Statistical results are shown in mean and standard error and *p* < 0.05 was considered statistically significant.

## 3. Results

### 3.1. Growth Performance

As shown in Table 1, compared with the CON group, the LAG and HAG groups had significantly increased 21 d body weight, 1–21 d ADG and 42 d body weight (*p* < 0.05) and the LAG group had significantly increased 22–42 d ADG (*p* < 0.05). For the whole feeding period (1–42 days), LAG and HAG groups had significantly increased ADG and significantly decreased FCR (*p* < 0.05). No significant difference was observed among the ANT, LAG and HAG groups (*p* > 0.05).

### 3.2. Apparent Metabolic Rate

As shown in Table 2, compared with the CON group, the LAG and HAG groups had significantly increased apparent metabolic rate of energy (*p* < 0.05) and the LAG group had significantly increased apparent metabolic rate of the CP (*p* < 0.05), No significant difference was observed among the ANT, LAG and HAG groups (*p* > 0.05). No significant differences were observed in the apparent metabolic rates of EE, Ca and P among the four groups (*p* > 0.05).

### 3.3. Meat Quality

As shown in Table 3, compared with the CON group, the LAG group had significantly increased pH_45min_, pH_24h_ and L* values (*p* < 0.05) and significantly decreased b* value, shear force and drip loss (*p* < 0.05), while the HAG group had significantly increased L* value (*p* < 0.05) and significantly decreased b* value (*p* < 0.05). Compared with the ANT group, the LAG group had significantly increased pH_45min_ and L* values (*p* < 0.05) and significantly decreased b* value and shear force (*p* < 0.05) and the HAG group had significantly decreased b* value (*p* < 0.05). Compared with the HAG group, the LAG group had significantly increased pH_45min_ and significantly decreased shear force (*p* < 0.05). No treatment difference was observed in the a* values (*p* > 0.05).

### 3.4. Breast Muscle Fatty Acid

As shown in Table 4, compared with the CON group, LAG and HAG groups had significantly decreased C22:0, total SFA and n-6/n-3 (*p* < 0.05) and significantly increased C16:1, C18:1n-9c, C24:1, C18:2n-6, C18:3n-3, C20:2, C22:6n-3, total MUFA, total PUFA and PUFA/SFA (*p* < 0.05). Compared with the ANT group, LAG and HAG groups had significantly increased C24:1, C18:3n-3, C20:2 and C22:6n-3 (*p* < 0.05) and significantly decreased n-6/n-3 (*p* < 0.05). Compared with the HAG group, the LAG group had significantly increased C16:1 and C18:1n-9c and significantly increased C18:3n-3 and C22:6n-3 (*p* < 0.05).

### 3.5. Serum and Breast Muscle Antioxidant Function

As shown in Table 5, compared with the CON group, LAG and HAG groups had significantly increased T-AOC activity in serum and breast muscle and SOD activity in serum (*p* < 0.05) and significantly decreased MDA content in serum and breast muscle (*p* < 0.05). Compared with the ANT group, LAG and HAG groups had significantly increased T-AOC activity in serum and breast muscle and significantly decreased MDA content (*p* < 0.05) and the LAG group had significantly increased serum GSH-Px and SOD activities (*p* < 0.05). The serum GSH-Px and SOD activities of the LAG group were significantly increased compared with those of the HAG group (*p* < 0.05). No treatment difference was observed in SOD and GSH-Px activities in the breast muscles (*p* > 0.05).

### 3.6. Intestine Antioxidant Enzyme mRNA Expression

**Figure 1:**
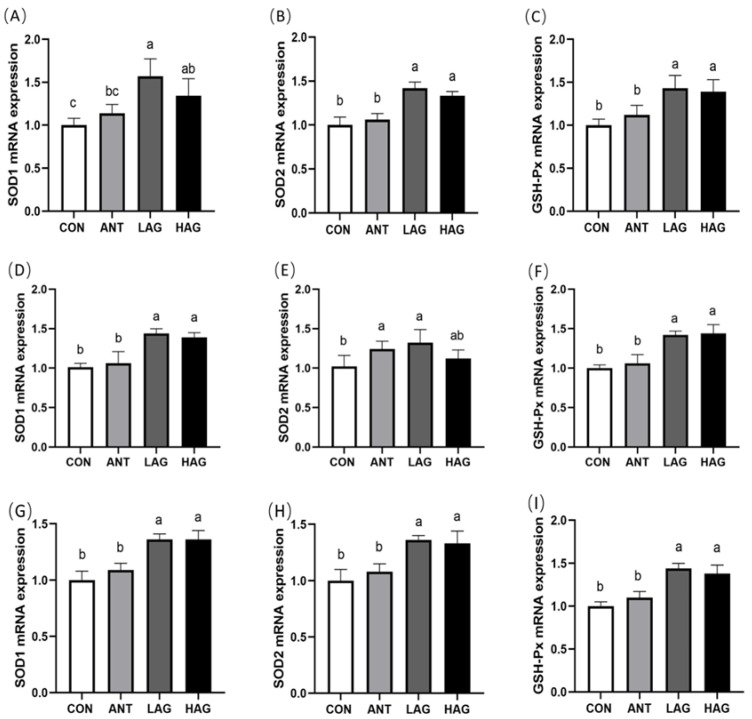
(**A**–**I**) Effect of Astragalus-Glycyrrhiza polysaccharides on mRNA expression of antioxidant enzyme-related in the duodenum, jejunum and ileum of broilers. Data are means ± SEM, n = 6. Means with different letters indicate significant differences (*p* < 0.05).

As shown in Figure 1A–C, compared with the CON group, the expression levels of *SOD1*, *SOD2* and *GSH-Px* mRNA in the duodenum of LAG and HAG groups were significantly increased (*p* < 0.05). Compared with the ANT group, the LAG and HAG groups had significantly increased expression levels of *SOD2* and *GSH-Px* mRNA (*p* < 0.05) and the LAG group had significantly increased *SOD1* mRNA expression (*p* < 0.05). No significant difference was observed between the LAG and HAG groups (*p* > 0.05).

As shown in Figure 1D–F, compared with the CON group, the LAG and HAG groups had significantly higher *SOD1* and *GSH-Px* mRNA expression levels in the jejunum (*p* < 0.05) and the LAG group had significantly higher *SOD2* mRNA expression (*p* < 0.05). Compared with the ANT group, the expression levels of *SOD1* and *GSH-Px* mRNA in the LAG and HAG groups were significantly increased (*p* < 0.05). No significant difference was observed between the LAG and HAG groups (*p* > 0.05).

As shown in Figure 1G–I, compared with the CON and ANT groups, LAG and HAG groups had significantly higher *SOD1, SOD2* and *GSH-Px* mRNA expression levels in the ileum (*p* < 0.05). No significant difference was observed between the LAG and HAG groups (*p* > 0.05).

### 3.7. Cecal Microbial Diversity

The Illumina Miseq high-throughput sequencing platform sequenced the V3-V4 region of the 16S rRNA gene. After removing incorrect chimeric sequences, 1,144,417 high-quality reads were generated. An average of 71,526 sequences was obtained per sample with an average length of 421 bp. As shown in Supplementary Figure S1A, the Shannon dilution curve reflected the sample’s microbial diversity index. In this experiment, the curves of each group tended to be flat, indicating that the amount of sequencing data was large enough and the sequencing results were reasonable. The Shannon index of the LAG group was the highest, indicating that the LAG group had the highest microbial diversity. Venn plots could count the number of common and unique operational taxonomic units (OTUs) in multiple samples. As shown in Supplementary Figure S1B, a total of 743 OTUs were shared by four groups and the unique OTUs of each group followed the order: LAG group (4208) > HAG group (4140) > CON group (2909) > ANT group (2785).

#### 3.7.1. Alpha Diversity Analysis

**Figure 2:**
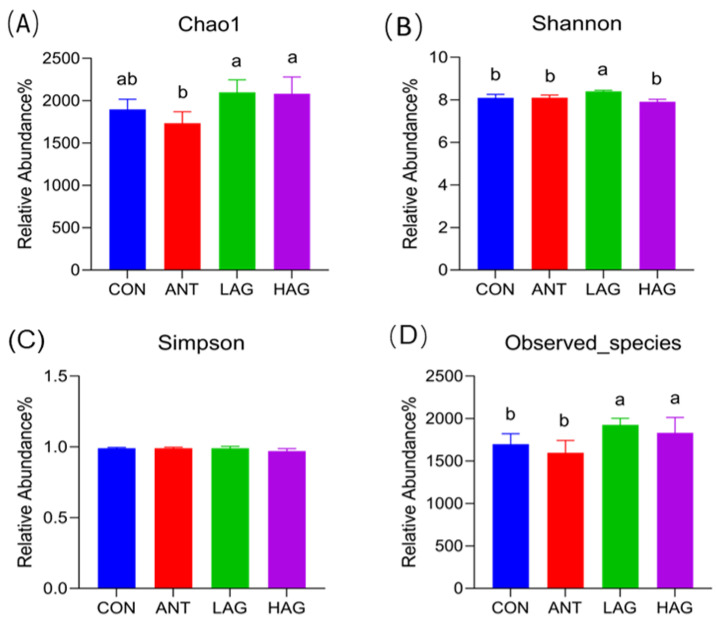
(**A**–**D**)**.** Effects of Astragalus-Glycyrrhiza polysaccharides on alpha diversity index of cecal microbiota in broilers. Means with different small letters indicate significant differences (*p* < 0.05).

The alpha diversity includes the Chao1 index, Shannon index, Simpson index and Observed species, which refers to the diversity within a specific area or ecosystem. The Chao index and Observed species are used to evaluate the richness of the microbiota. A higher Chao or Observed species indicates a higher richness of the microbiota. A higher Simpson index indicates a low microbiota diversity. The data of alpha diversity indexes are presented in Figure 2A–D. Compared with the CON group, the Shannon index and Observed species in the LAG group and the Observed species in the HAG group were significantly increased (*p* < 0.05). Compared with the ANT group, the Chao1 index, Shannon index and Observed species in the LAG group (*p* < 0.05) and the Chao1 index and Observed species in the HAG group (*p* < 0.05) were significantly increased. Compared with the HAG group, the Shannon index in the LAG group was significantly increased (*p* < 0.05). No significant difference in the Simpson index was observed among the groups (*p* > 0.05).

#### 3.7.2. Beta Diversity Analysis

**Figure 3:**
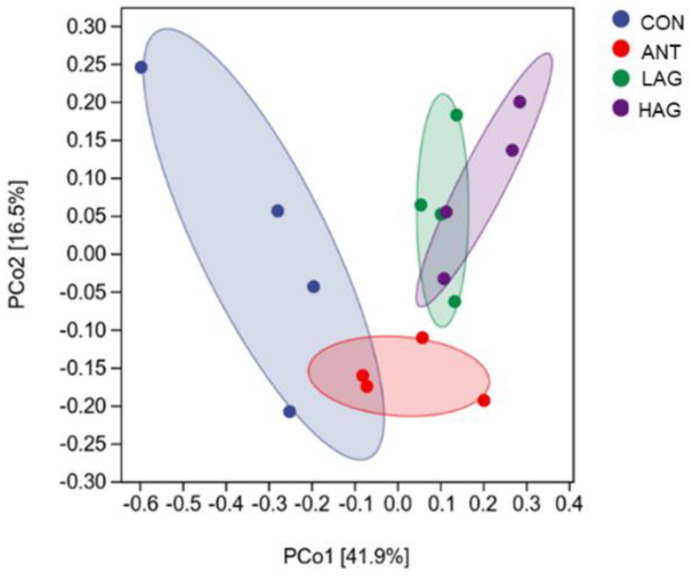
The cecal microbiota’s principal coordinate analysis (PCoA) was based on the Weighted UniFrac distance (n = 4).

Beta diversity was used to analyze the similarity of cecal microbiota among different groups. The differences or similarities of cecal microbial diversity among groups were comprehensively analyzed by the principal coordinates analysis (PCoA) based on the Weighted UniFrac distance. As shown in Figure 3, the distances of the samples in the CON group were scattered, indicating that the samples in this group had poor uniformity. LAG and HAG groups were wholly separated from the CON and ANT groups, indicating that LAG and HAG groups changed the bacterial community structure. A crossover was observed between LAG and HAG groups, suggesting a similarity in the structures of their cecal microbiota.

#### 3.7.3. Microflora Structure

**Figure 4:**
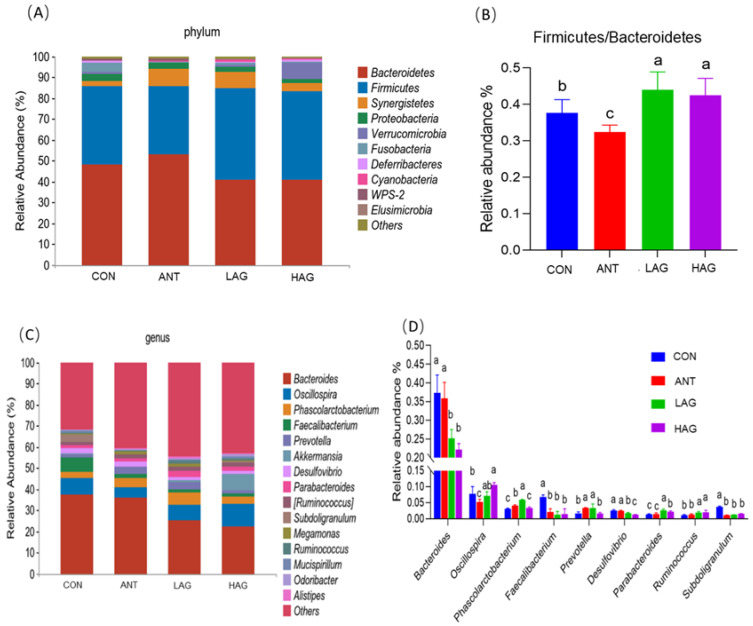
Effect of Astragalus-Glycyrrhiza polysaccharides on the abundance of cecal microflora at phylum and genus level in broilers (**A**) Relative abundance of cecal microbiota at the phylum level; (**B**) Comparison of Firmicutes and Bacteroidetes ratio. (*p* < 0.05). (**C**) Relative abundance of cecal microbiota at the genus level; (**D**) Relative abundance difference analysis of cecal bacterial species at the genus level (*p* < 0.05). Means with different small letters indicate significant differences (*p* < 0.05).

At the phylum level, the effects of *Astragalus- Glycyrrhiza* polysaccharides on the cecal microbial composition of broilers are shown in Figure 4A,B. The dominant phyla of the four groups were *Bacteroidetes* and *Firmicutes*. CON, ANT, LAG and HAG had relative abundance values of *Bacteroidetes* of 49.12%, 54.15%, 40.87% and 40.91%, respectively and relative abundance values of *Firmicutes* of 37.67%, 32.42%, 43.97%, 42.49%, respectively. Compared with the CON and ANT groups, LAG and HAG groups had increased the relative abundance of *Firmicutes* and the ratio of *Firmicutes* and *Bacteroidetes*(F/B) decreased the relative abundance of *Bacteroidetes*. At the genus level, the effect of *Astragalus-Glycyrrhiza* polysaccharides on the cecal microbial composition of broilers was shown in Figure 4C,D. The dominant genera of the four groups were *Bacteroides, Oscillospira, Phascolarctobacterium* and *Faecalibacterium*. Figure 4D showed the significant difference in the relative abundance of 9 of the top 15 genera among the four treatment groups.

Compared with the CON and ANT groups, LAG and HAG groups increased the relative abundance of *Oscillospira, Parabacteroides* and *Ruminococcus* while decreased the abundance of *Bacteroides, Faecalibacterium, Desulfovibrio* and *Subdoligranulum*. In addition, the LAG group increased the relative abundance of *Phascolarctobacterium.*

### 3.8. Serum Metabolomic Analysis

Through the analysis of apparent performance and gut microbiota, we found that the production performance of the LAG group was better than that of HAG. Furthermore, CON and LAG were used to analyze serum metabolites. Orthogonal partial least squares discriminant analysis (OPLS-DA) was used to distinguish the difference between groups in positive and negative ion mode. Response permutation testing (RPT) was used to evaluate the accuracy of the OPLS-DA model. R^2^Ywas closer to 1, the more stable and reliable the model; Q^2^Y was below 0.05 and represented a credible predictive ability. In this experiment, R^2^Y was above 0.7 and Q^2^Ywas below 0.05, indicating a good model prediction ability. (Supplementary Figure S2). Further OPLS-DA analysis of the data was performed in positive and negative ion modes; the samples in the same group were clustered closely, indicating that their metabolites were highly similar and stable and the samples in different groups were wholly separated, indicating that there were significant differences in serum metabolites between the CON and LAG group.

**Figure 5:**
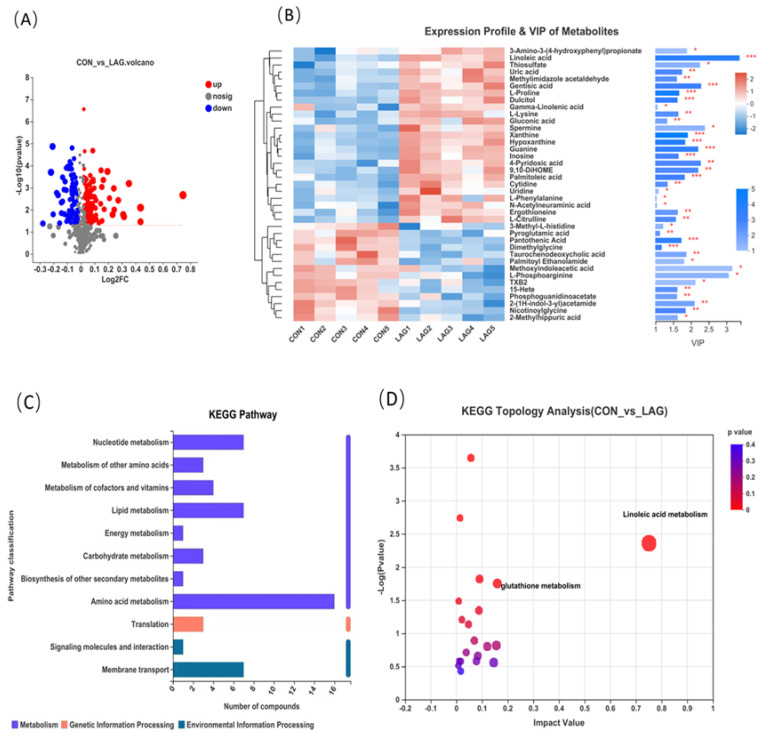
(**A**) Differential volcano plot of CON and LAG groups, each point in the graph represents a specific metabolite and larger points indicate higher VIP values. Blue indicates significant downregulation and red indicates significant upregulation. (**B**) Heatmap combined with hierarchical clustering of the most significantly influenced metabolites in CON and LAG groups. Only the metabolites with the highest variable importance in projection (VIP) value in the OPLS-DA model (top 40 named metabolites) were listed. The *t*-test was jointly applied with the OPLS-DA to identify the discrepant metabolites and the *p* value in the *t*-test was shown. The heatmap colors represent the relative expression of metabolites in the sample, with VIP bar graphs of metabolites on the right. The bars’ length represents the metabolites’ contribution value to the difference between the two groups. The larger the value, the more significant the difference between the two groups. The bar’s color indicates the significance of the metabolite difference between the two groups (* 0.01 < *p* ≤ 0.05, ** 0.001 < *p* ≤ 0.01, *** *p* ≤ 0.001). (**C**) KEGG pathway, the ordinate, is the second classification of the KEGG metabolic pathway and the abscissa is the number of metabolites annotated to the pathway. (**D**) KEGG topology analysis. Each bubble in the figure represents a KEGG Pathway; the horizontal axis represents the relative importance of the metabolites in the pathway and the size of the Impact Value; the vertical axis represents the enrichment significance of metabolites involved in the pathway-log10 (*p*-value); bubbles of the size represents the Impact Value; the larger the bubble, the greater the importance of the pathway.

As shown in Figure 5A differential volcanic plot, a total of 193 differential metabolites were identified in the two treatments, of which 113 differential metabolites were significantly up-regulated and 80 differential metabolites were significantly down-regulated (VIP ≥ 1.0, *p*-values < 0.05). Moreover, VIP in the OPLS-DA model was calculated to evaluate the changes in serum metabolites. Metabolites with VIP values > 1.0 and *p*-values < 0.05 (*t*-test) were considered significantly influenced by LAG supplementation and the top 40 metabolites with the highest VIP values were listed in Figure 5B, which revealed that serum metabolites among the two groups formed distinct clusters. Figure 5C shows that the identified metabolites were functionally annotated through the KEGG database. The annotated metabolites were mainly focused on the processes of amino acid metabolism, lipid metabolism, nucleotide metabolism, membrane transport, etc. Furthermore, as shown in Figure 5D, pathway enrichment and topology analysis were performed.

The identified metabolites with an impact value higher than 0.1 and *p*-values below 0.05 were mainly enriched in two pathways, namely the linoleic acid metabolism pathway (Impact Value = 0.75, *p* = 0.004) and glutathione metabolism pathway (Impact Value = 0.16, *p* = 0.018). Differential metabolites from the linoleic acid metabolism pathway, including linoleic acid and gamma-linolenic acid and the glutathione metabolism pathway includes two differential metabolites, spermine and pyroglutamic acid.

### 3.9. Correlation Analysis

**Figure 6:**
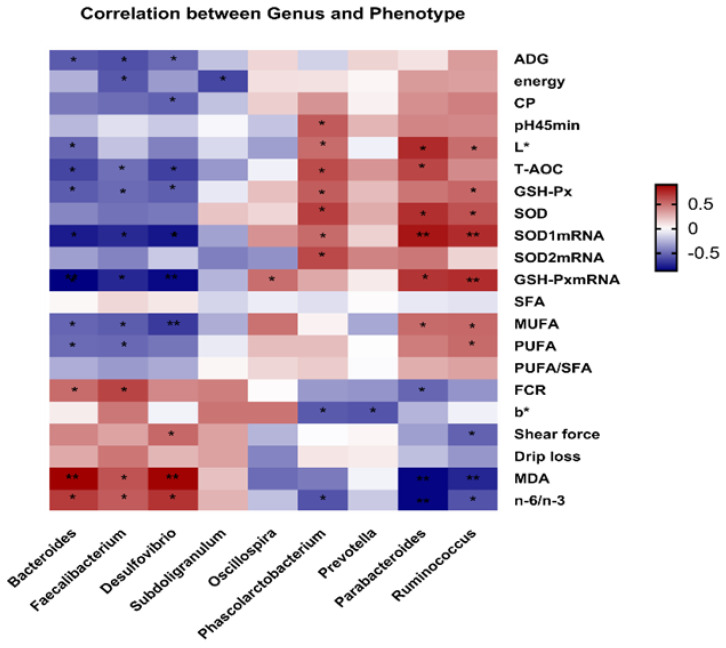
Correlation between the differential cecal microbial community of genera and the phenotype of broilers by Pearson correlation analysis. Red indicates positive correlation and blue indicates negative correlation. (* 0.01 < *p* ≤ 0.05, ** 0.001 < *p* ≤ 0.01).

In order to predict the correlation between the top nine differential bacterial genera and 21 differential phenotypic indicators, a Pearson correlation analysis was conducted. As shown in Figure 6, *Bacteroides* was positively correlated with n-6/n-3, MDA and FCR but negatively correlated with ADG, L*, T-AOC, GSH-Px, *SOD1* mRNA expression, *GSH-Px* mRNA expression, MUFA and PUFA (*p* < 0.05).

*Faecalibacterium* was positively correlated with n-6/n-3, MDA and FCR but negatively correlated with ADG, energy, T-AOC, GSH-Px, *SOD1* mRNA expression, *GSH-Px* mRNA expression, MUFA and PUFA (*p* < 0.05). *Desulfovibrio* was positively correlated with n-6/n-3, MDA and shear force but negatively correlated with ADG, CP, T-AOC, GSH-Px, *SOD1* mRNA expression, *GSH-Px* mRNA expression and MUFA (*p* < 0.05). *Subdoligranulum* is only negatively correlated with energy. Similarly, *Oscillospir* was only positively correlated with *GSH-Px* mRNA expression (*p* < 0.05). *Phascolarctobacterium* was positively correlated with pH 45 min, L*, T-AOC, GSH-Px, SOD, *SOD1* mRNA expression and *SOD2* mRNA expression, but negatively correlated with n-6/n-3 and b* (*p* < 0.05). *Prevotella* was only negatively correlated with b* (*p* < 0.05). *Parabacteroides* was positively correlated with L*, T-AOC, SOD, *SOD1* mRNA expression, *GSH-Px* mRNA expression and MUFA but negatively correlated with FCR, MDA and n-6/n-3 (*p* < 0.05). *Ruminococcus* was positively correlated with L*, GSH-Px, SOD, *SOD1* mRNA expression, *GSH-Px* mRNA expression, MUFA and PUFA, but negatively correlated with shear force, MDA and n-6/n-3 (*p* < 0.05). These results indicated a specific correlation between the bacterial genera and phenotypic indicators.

**Figure 7:**
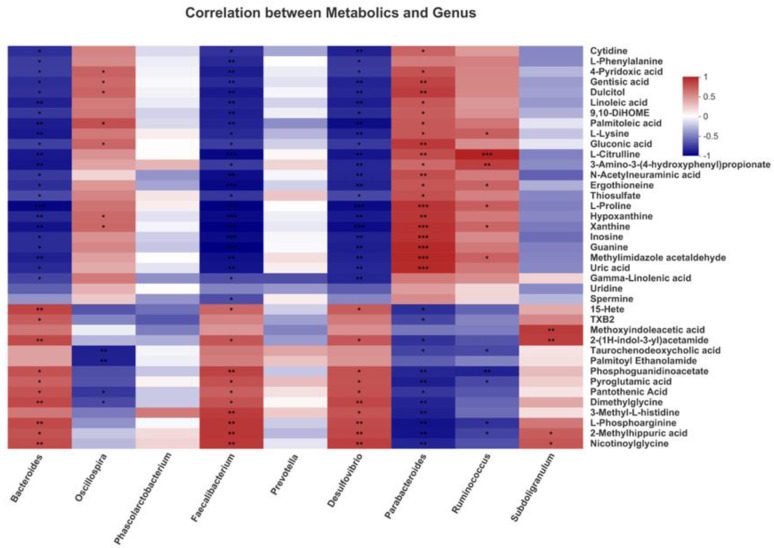
Correlation between the differential cecal microbial community of genera and the serum metabolites of broilers by Pearson correlation analysis. Red indicates positive correlation and blue indicates negative correlation. (* 0.01 < *p* ≤ 0.05, ** 0.001 < *p* ≤ 0.01, *** *p* ≤ 0.001).

Since two critical metabolic pathways, linoleic acid metabolism and glutathione metabolism, were found in metabolites pathway enrichment analysis, we investigated the correlation of 9 differential bacterial genera with four differential metabolites enriched in these two pathways by Pearson’s correlation analysis. The heatmap revealed that nine species were correlated with the top 40 metabolites (Figure 7).

Differential metabolites from the linoleic acid metabolism pathway, including linoleic acid and gamma-linoleic acid, negatively correlated with *Bacteroides*, *Faecalibacterium* and *Desulfovibrio*; moreover, linoleic acid was positively correlated with *Parabacteroides* (*p* < 0.05). Differential metabolites from the glutathione metabolism pathway, including pyroglutamic acid and spermine. Pyroglutamic acid was positively correlated with *Bacteroides*, *Faecalibacterium* and *Desulfovibrio* but negatively correlated with *Parabacteroides* and *Ruminococcus*; moreover, spermine was positively correlated with *Faecalibacterium* (*p* < 0.05). In addition, many differential metabolites such as gluconic acid, L-proline and nicotinylglycine were significantly correlated with certain bacteria such as *Bacteroides*, *Faecalibacterium* and *Desulfovibrio* (*p* < 0.05). These results suggested that LAG-mediated change of certain bacteria were correlated with metabolites in linoleic acid metabolism and glutathione metabolism, indicating the critical roles of these bacteria in LAG-associated beneficial effects.

**Figure 8:**
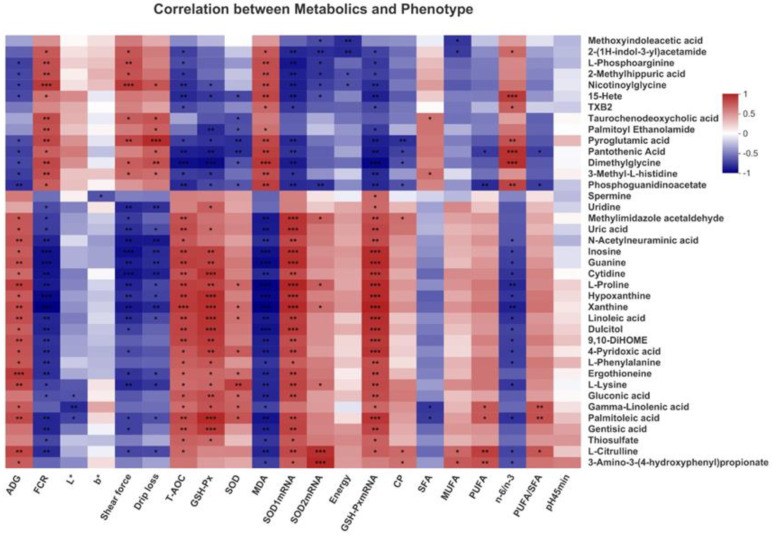
Correlation between the differential serum metabolites and the phenotype of broilers by Pearson correlation analysis. Red indicates positive correlation and blue indicates negative correlation. (* 0.01 < *p* ≤ 0.05, ** 0.001 < *p* ≤ 0.01, *** *p* ≤ 0.001).

Correlation analysis of the top 40 differential serum metabolites and 21 differential phenotypic indicators showed that in Figure 8.

Linoleic acid and gamma-linoleic acid were positively correlated with ADG, T-AOC, GSH-Px, *SOD1* mRNA and *GSH-Px* mRNA but negatively correlated with FCR and MDA (*p* < 0.05). Gamma-linoleic acid was positively correlated with SOD, PUFA and PUFA/SFA but negatively correlated with SFA (*p* < 0.05). Conversely, Pyroglutamic acid was positively correlated with FCR, shear force, drip loss, MDA and n-6/n-3 but negatively correlated with ADG, T-AOC, GSH-Px, SOD, *SOD1*mRNA and *GSH-Px* mRNA (*p* < 0.05). Spermine was positively correlated with *GSH-Px* mRNA but negatively correlated with b*. In addition, many differential metabolites such as inosine, guanine and cytidine were significantly correlated with phenotypic indicators such as T-AOC, GSH-Px, SOD, *SOD1*mRNA and *GSH-Px* mRNA (*p* < 0.05). These results suggested that specific phenotypic indicators that LAG changed were correlated with metabolites in linoleic acid metabolism and glutathione metabolism, indicating the critical roles of these indicators in LAG-associated beneficial effects.

## 4. Discussion

Under the comprehensive “ban on antibiotics”, exploring “new, efficient, safe and green” antibiotic substitutes has gradually become a research hotspot in the feed industry. Several studies showed that adding plant extracts to poultry diets can improve poultry immunity, prevent disease and promote poultry growth [6,23]. Adding APS to poultry diets can increase ADFI, reduce FCR [14] and alleviate growth decrease under cyclophosphamide and lipopolysaccharide stress in broilers [15,16]. Adding 10 g/kg of *Astragalus* powder to the diet can increase the ADG and reduce the FCR of broilers [24]. Drinking water containing *Glycyrrhiza* extract could improve body weight, ADFI and ADG and reduce FCR in broilers under heat stress [25]. Adding 500 mg/kg of Glycyrrhiza extract to high-density broilers diets effectively improves weight gain in late growth and throughout the growth period [26]. Our experiment showed that LAG and HAG supplementation increased body weight and ADG and decreased FCR. In addition, linoleic acid and gamma-linoleic acid were positively correlated with ADG but negatively correlated with FCR; conversely, pyroglutamic acid was positively correlated with FCR, indicating that the addition of *Astragalus-Glycyrrhiza* polysaccharide could improve the growth performance of broilers by altering the linoleic acid metabolism and glutamate metabolism pathways.

The nutrient metabolic rate is an important indicator to measure the digestion and absorption of nutrients by animals. Its level directly affects the growth performance of animals and reflects the diet’s nutritional value. Several studies have shown that dietary supplementation with APS can improve the metabolism of energy and CP [16]. Ibrahim et al. [27] showed that adding 3% *Glycyrrhiza glabra* extract residue to the broiler diet can increase CP’s apparent metabolic rate and decrease fat’s apparent metabolic rate. Our experiment showed that LAG and HAG groups increased broiler diets’ apparent metabolic energy rate. Moreover, the LAG group achieved a higher apparent metabolic rate of CP than antibiotics. It speculated that the improvement of nutrient apparent metabolic rate might be related to the improvement of production performance, further indicating that the *Astragalus-Glycyrrhiza* polysaccharides have a specific promotion effect on the growth and feed utilization of broilers.

Muscle pH, shear force and drip loss are indicators for evaluating the physicochemical properties of meat quality. The decrease in pH after animal slaughter is related to the glycolysis of muscle glycogen. Stress accelerates glycogenolysis in the body and causes the rapid decrease of muscle’s pH, seriously affecting muscle’s color and taste [28]. Galli et al. [29] showed that adding microencapsulated organic acids to broiler diets can reduce the rate of glycolysis and pH decline and improve meat quality. A high pH_24h_ results in a low shear force, high water-holding capacity and improved meat quality [30]. Studies showed that the fermented *Astragalus-Glycyrrhiza* water extract as a feed additive could reduce drip loss of breast and leg muscles [31]. Our experiment found that the LAG group increases broilers’ pH_45min_ and pH_24h_ and reduces drip loss and shear force, indicating that the moderate addition of *Astragalus-Glycyrrhiza* polysaccharides could effectively alleviate the glycolysis post-slaughter and maintain the juiciness of the meat. This finding shows that the *Astragalus-Glycyrrhiza* polysaccharides are better at improving meat quality than antibiotics.

The color of meat, the most intuitive external expression for consumers to evaluate the freshness of meat, is usually expressed by L*, a* and b* values. The L* value indicates the muscle’s oxidized myoglobin content and the normal range is 46–53. Within this range, a higher value represents improved gloss. The a* value represents the content of deoxy-myoglobin in the muscle and a higher value indicates improved meat color and fresh meat. The b* value reflects the content of oxidized methemoglobin and a lower value indicates fresh meat [26,32,33]. Alagawany et al. [21] showed that drinking fermented *Astragalus-Glycyrrhiza* water extract improved the a* value and decreased the b* value and drip loss in broilers. Our experiment showed that the L* value was within the normal range and LAG and HAG supplementation increased the L* value and decreased the b* value, indicating that the addition of *Astragalus-Glycyrrhiza* polysaccharides helps in improving the freshness of broiler meat.

Chicken has become a popular functional food for consumers because of its high protein, low cholesterol and low saturated fatty acids [34]. The fatty acid content in chicken primarily consists of unsaturated fatty acids, such as linoleic acid (C18:2n-6), linolenic acid (C18:3n-3) and arachidonic acid (C20:4) is an essential precursor of chicken flavor [35]. Our results showed that LAG and HAG groups increased the contents of C18:2n-6 and C18:3n-3 in chicken and the content of total PUFA, thereby improving the flavor of the meat. Studies showed that imbalances in the ratios of PUFA to SFA and PUFA n-6 to PUFA n-3 are associated with various diseases, such as cardiovascular disease, inflammatory disease, diabetes and autoimmune diseases [36,37]. Hu et al. [38] showed that high PUFA n-3 and low SFA contents can improve meat quality and nutritional value, thereby reducing the risk of cardiovascular disease. PUFA n-3 has excellent anti-aging, anti-inflammatory, antioxidant, anti-cancer, anti-arthritis, anti-depressant, anti-hypertensive and insulin-sensitizing effects and cardiovascular health benefits [39]. The present study showed that LAG and HAG groups increased the ratio of PUFA to SFA and decreased the ratio of PUFA n-6 to PUFAn-3. In addition, gamma-linoleic acid was positively correlated with PUFA and PUFA/SFA but negatively correlated with SFA, indicating that the addition of *Astragalus-Glycyrrhiza* polysaccharide could improve meat quality by changing the linoleic acid metabolic pathway.

GSH-Px, SOD, T-AOC and MDA are indicators used to measure the body’s antioxidant capacity. Studies showed that APS could scavenge free radicals in time by activating various enzyme activities in the body, reducing oxidative stress in animals and enhancing animal immune responses [40]. The present experiment showed that LAG and HAG groups increased the expression levels of *SOD1* mRNA, *SOD2* mRNA and *GSH-Px* mRNA in the small intestine (duodenum, jejunum and ileum) of broilers. Moreover, the LAG supplementation increased serum T-AOC, SOD, GSH-Px and breast muscle’s T-AOC activity. At the same time, it decreased the MDA content in serum and breast muscle. Similar results were reported by Wu et al. [13] who found that adding the diet with 0.5–1.0 g/kg APS could improve the growth performance and serum SOD, GSH-Px, IgG, IgM and IgA and reduce the MDA content in broilers. Adding Astragalus root powder can enhance broilers’ growth performance, antioxidant status and serum metabolites and improve liver and kidney functions by improving the antioxidant status [41,42]. Adding the *Glycyrrhiza* extract to chicken patties reduces the production of MDA and increases the pH and a* values in the patties, thereby improving the oxidative stability of chicken patties and prolonging the shelf life [43].

The change of gut microbiome disrupts gut function when broilers are immunosuppressed by environmental stress or viral infection [44]. Studies showed that some plant polysaccharides reaching the distal gastrointestinal tract could be fermented by the gut microbiota and further regulate the gut microenvironment [45]. Broilers fed with γ-irradiated Astragalus polysaccharides show higher bacterial OTUs and the Shannon index but decrease the Simpson index than control and cyclophosphamide-treated groups [46]. Consistent with these findings, our study showed that LAG and HAG had more bacterial OTUs than the CON and ANT groups. Moreover, the result of Alpha diversity revealed that LAG and HAG groups increased the Chao1 index and Observed species. Beta diversity results showed that LAG and HAG groups altered the gut microbiota structure and composition, indicating that dietary supplementation with *Astragalus-Glycyrrhiza* polysaccharide increased the richness and diversity of cecal microbiota of broilers.

The dominant phyla in this experiment are *Bacteroidetes* and *Firmicutes*. Many bacteria belonging to *Firmicutes* are involved in energy metabolism and maintenance of intestinal health [47]. An increase in F/B favors nutrient absorption and is closely related to gut microbiota composition and the ability of the host to acquire energy [48,49]. The present experimental study showed that adding *Astragalus-Glycyrrhiza* polysaccharides increased F/B, which was beneficial to the absorption of nutrients by intestinal microorganisms in broilers.

At the genus level, the dominant genera with significant differences in this experiment are *Bacteroides*, *Oscillospira*, *Phascolarctobacterium* and *Faecalibacterium*. *Bacteroides* is one of the predominant genera of anaerobic bacteria in the chicken cecum [50]. Previous studies have shown that *Bacteroides* was positively related to serum inflammatory cytokines TNF-a, IL-1b and IL-6 and dietary supplementation with APS and GPS could inhibit the proliferation of *Bacteroides* [20]. Liu et al. [46] showed that the abundance of *Bacteroides* in broilers fed with γ-irradiated *Astragalus* polysaccharides was lower than those in the cyclophosphamide-treated groups. These findings are consistent with the results of the present experiment that LAG and HAG supplementation reduce the abundance of *Bacteroides* and *Bacteroides* were positively correlated with MDA but negatively correlated with T-AOC GSH-Px, *SOD1* mRNA expression and *GSH-Px* mRNA expression. It has been reported that *Oscillospira* could produce butyrate by gluconate and human health is positively correlated with *Oscillospira* [51,52]. The supplementation of probiotic preparations and *Bacillus subtilis* in laying hen diets increases the abundance of *Oscillospira*, decreases the abundance of pathogenic *E. coli* and improves the performance and intestinal function in laying hens [53]. Our experiment showed that *Oscillospira* was positively correlated with *GSH-Px* mRNA expression and LAG and HAG supplementation increased the abundance of *Oscillospira*. Hou et al. [54] found that the abundance of *Phascolarctobacterium* in the gut microbiota in patients with ulcerative colitis was remarkably reduced compared with that in healthy individuals. Plantain is a widely used remedy for constipation. Jalanka et al. [55] found that plantain could increase the abundance of *Phascolarctobacterium* in the fecal microorganisms of constipated patients and reduce constipation. In this experiment, LAG supplementation increased the relative abundance of *Phascolarctobacterium* while *Phascolarctobacterium* was positively correlated with T-AOC, GSH-Px, SOD, *SOD1* mRNA expression and *SOD2* mRNA expression. Maier et al. [56] reported that enhancing the activity of *Faecalibacterium* does not enhance the integrity of the intestinal barrier. Liu et al. [46] found that APS supplementation significantly decreased the abundance of *Faecalibacterium* in broilers. In our experiment, LAG and HAG supplementation decrease the relative abundance of *Faecalibacterium*. *Faecalibacterium* was positively correlated with MDA but negatively correlated with T-AOC, GSH-Px, SOD1 mRNA expression and GSH-Px mRNA expression, indicating that the *Astragalus-Glycyrrhiza* polysaccharides could improve antioxidant function by modulating gut microbiota in broilers.

Metabolomics is widely utilized to investigate the impact of changes in animal diet, environment, genes and gut microbial communities on the response pathways of metabolic systems [57]. In this study, untargeted metabolomics of broiler serum were analyzed by LC-MS. Through the OPLS-DA model analysis, it was found that there was a significant difference between LAG and CON groups, indicating that the addition of *Astragalus-Glycyrrhiza* polysaccharides could significantly affect the metabolism of broiler serum. A total of 193 significant differential metabolites were identified for both groups, of which 113 differential ions were significantly up-regulated and 80 differential ions were significantly down-regulated. Through the KEGG database, the identified metabolites were functionally annotated. Amino acid metabolism, lipid metabolism, nucleotide metabolism and membrane transport are these compounds’ primary biological metabolic and signal transduction processes. Furthermore, the pathway of linoleic acid metabolism and glutathione metabolism pathway were identified as the most significant pathway through KEGG topology analysis.

Linoleic acid belongs to omega-6 PUFA and is essential for normal growth and development, cell function and signal transduction and immune response [58]. Linoleic acid can be elongated and desaturated to other bioactive omega-6 PUFAs, such as gamma-linolenic acid (18:3n6) and arachidonic acid (20:4n6). Subsequently, arachidonic acid can be converted to bioactive compounds, such as prostaglandins and leukotrienes. These eicosanoids are important in the normal metabolic function of tissues and cells [59]. Studies have shown that supplementing 1% linoleic acid in parental pigeon diets could improve the health of pigeons by increasing antioxidant capacity and lipid metabolism [60]. Linoleic acid could also improve antioxidative capacity in laying hens [61] and lipid metabolism in broilers and ducks [62,63]. The high intake of linoleic acid is associated with reduced risk for heart diseases and type 2 diabetes [64]. Linoleic acid induced autophagy and increased antioxidant ability through the adenosine monophosphate-activated protein kinase (AMPK) signaling pathway and the AMPK-target of rapamycin (TOR) signaling pathway in hepatocytes in vitro and could greatly aid in the prevention and treatment of multiple pathologies [65]. In our study, LAG supplementation elevated linoleic acid levels and gamma-linolenic acid levels. T-AOC, GSH-Px, SOD and GSH-Px mRNA were positively correlated with these two metabolites, indicating that LAG could improve antioxidant capacity by modulating linoleic acid metabolism. In addition, *Parabacteroides* was positively correlated with linoleic acid. Conversely, *Bacteroides*, *Faecalibacterium* and *Desulfovibrio* were negatively correlated with linoleic acid and gamma-linolenic acid, suggesting that these species might participate in the modulation of linoleic acid metabolism by LAG.

The glutathione metabolism pathway includes two differential metabolites, spermine and pyroglutamic acid, which were changed by LAG. Spermine and pyroglutamic acid are intermediates in glutathione metabolism. Glutathione plays a critical role in protecting cells from oxidative damage and the toxicity of xenobiotic electrophiles and maintaining redox homeostasis [66]. Studies have shown that elevated levels of pyroglutamic acid are associated with impaired glutathione metabolism [67,68]. In our study, LAG supplementation reduced the levels of pyroglutamic acid and T-AOC, GSH-Px, SOD, *SOD1mRNA* and *GSH-Px mRNA* were negatively correlated with pyroglutamic acid, indicating the protective effect of LAG supplementation on glutathione metabolism. In addition, *Bacteroides*, *Faecalibacterium* and *Desulfovibrio* were positively correlated with pyroglutamic acid. Conversely, *Parabacteroides* and *Ruminococcus* were negatively correlated with pyroglutamic acid. *Faecalibacterium* was positively correlated with spermine, suggesting that these species might participate in the modulation of glutathione metabolism by LAG.

## 5. Conclusions

This study showed that dietary supplementation with 150 mg/kg *Astragalus* polysaccharides and 75 mg/kg *Glycyrrhiza* polysaccharides or 300 mg/kg *Astragalus* polysaccharides and 150 mg/kg *Glycyrrhiza* polysaccharides could improve production performance, antioxidant function, meat quality and modulate the diversity, richness and composition of cecal microbiota of broilers, which achieved the effects of Terramycin calcium. In addition, the metabolomics analysis showed that supplementation with 150 mg/kg *Astragalus* polysaccharides and 75 mg/kg *Glycyrrhiza* polysaccharides screened some differential metabolites, mainly concentrated in the linoleic acid metabolism and glutathione metabolism pathway. Correlation analysis showed that production performance, antioxidant function and meat quality significantly correlated with cecal microbiota and serum metabolites. Overall, these findings indicate that dietary supplementation with 150 mg/kg *Astragalus* polysaccharides and 75 mg/kg *Glycyrrhiza* polysaccharides could improve production performance, antioxidant function and meat quality by changing cecal microbiota and serum metabolites, which would further provide helpful information for developing effective and safe antibiotic alternatives in the poultry industry.

## Figures and Tables

**Table 1:** Effects of *Astragalus-Glycyrrhiza* polysaccharides on broiler performance.

	Treatments					
Items	CON	ANT	LAG	HAG	SEM	*p*-Value
**BW**						
Initial (g)	46.05	46.30	46.20	45.97	0.15	0.893
21 day (g)	841.72 ^b^	866.47 ^a^	885.78 ^a^	870.14 ^a^	5.05	0.010
42 day (g)	2490.91 ^b^	2581.66 ^a^	2657.41 ^a^	2585.83 ^a^	17.81	0.003
**Day1–21**						
ADFI (g/d)	48.16	50.20	47.24	46.56	0.67	0.244
ADG (g/d)	37.89 ^b^	39.06 ^a^	39.99 ^a^	39.23 ^a^	0.24	0.009
FCR (g·g)	1.27	1.28	1.18	1.19	0.02	0.121
**Day22–42**						
ADFI (g/d)	142.78	138.50	148.05	142.55	1.26	0.054
ADG (g/d)	78.53 ^b^	81.68 ^ab^	84.36 ^a^	81.70 ^ab^	0.73	0.029
FCR (g·g)	1.82	1.70	1.76	1.75	0.01	0.296
**Day1–42**						
ADFI (g/d)	95.11	94.52	97.64	94.55	0.58	0.168
ADG (g/d)	58.21 ^b^	60.37 ^a^	62.18 ^a^	60.47 ^a^	0.42	0.003
FCR (g·g)	1.63 ^a^	1.57 ^b^	1.57 ^b^	1.56 ^b^	0.01	<0.001

Note: The data were shown as means and standard error (SEM) (n = 6). The different lowercase letters in the same rows indicate significant difference (*p* < 0.05), while the same or without lowercase letters in the same rows indicate insignificant difference (*p* > 0.05). BW: Body weight; ADG: Average daily gain; ADFI: Average daily feed intake; FCR: Feed conversion rate; CON: basal diet group; ANT: supplement with 500 mg/kg oxytetracycline calcium; LAG: supplement with 150 mg/kg *Astragalus* polysaccharides and 75 mg/kg *Glycyrrhiza polysaccharides*; HAG: supplement with 300 mg/kg *Astragalus* polysaccharides and 150 mg/kg *Glycyrrhiza* polysaccharides.

**Table 2:** Effects of *Astragalus-Glycyrrhiza* polysaccharides on apparent metabolic rate of nutrients in broilers (%).

	Treatments					
Items	CON	ANT	LAG	HAG	SEM	*p*-Value
Energy	70.76 ^b^	74.77 ^a^	74.90 ^a^	75.49 ^a^	0.68	0.043
CP	47.47 ^b^	50.46 ^ab^	52.75 ^a^	49.98 ^ab^	0.63	0.019
EE	73.73	75.09	75.13	75.93	0.43	0.355
Ca	44.66	45.70	47.20	46.29	0.53	0.399
P	44.16	45.61	46.07	47.15	0.62	0.411

Note: The data were shown as means and standard error (SEM) (*n* = 6). The different lowercase letters in the same rows indicate significant difference (*p* < 0.05), while the same or without lowercase letters in the same rows indicate insignificant difference (*p* > 0.05). CP: crude protein; EE: crude fat; Ca: Calcium; P: phosphorus; CON: basal diet group; ANT: supplement with 500 mg/kg oxytetracycline calcium; LAG: supplement with 150 mg/kg *Astragalus* polysaccharides and 75 mg/kg *Glycyrrhiza polysaccharides*; HAG: supplement with 300 mg/kg *Astragalus* polysaccharides and 150 mg/kg *Glycyrrhiza* polysaccharides.

**Table 3:** Effects of *Astragalus-Glycyrrhiza* polysaccharides on meat quality.

	Treatments					
Items	CON	ANT	LAG	HAG	SEM	*p*-Value
pH_45min_	6.24 ^b^	6.24 ^b^	6.50 ^a^	6.24 ^b^	0.04	0.038
pH_24h_	5.75 ^b^	5.82 ^ab^	5.91 ^a^	5.83 ^ab^	0.02	0.032
L*	46.35 ^c^	49.51 ^b^	52.61 ^a^	51.81 ^ab^	0.65	<0.001
a*	6.79	6.29	6.93	5.69	0.28	0.423
b*	8.51 ^a^	8.65 ^a^	4.34 ^b^	6.32 ^b^	0.51	0.001
Shear force	27.14 ^a^	26.65 ^a^	24.34 ^b^	26.39 ^a^	0.39	0.047
Drip loss	1.62 ^a^	1.08 ^b^	0.96 ^b^	1.39 ^ab^	0.09	0.028

Note: The data were shown as means and standard error (SEM) (n = 6). The different lowercase letters in the same rows indicate significant difference (*p* < 0.05), while the same or without lowercase letters in the same rows indicate insignificant difference (*p* > 0.05). L*: brightness; a*: redness; b*: yellowness; CON: basal diet group; ANT: supplement with 500 mg/kg oxytetracycline calcium; LAG: supplement with 150 mg/kg *Astragalus* polysaccharides and 75 mg/kg *Glycyrrhiza polysaccharides*; HAG: supplement with 300 mg/kg *Astragalus* polysaccharides and 150 mg/kg *Glycyrrhiza* polysaccharides.

**Table 4:** Effects of *Astragalus-Glycyrrhiza* polysaccharides on the fatty acid composition of breast muscle.

	Treatments					
Items	CON	ANT	LAG	HAG	SEM	*p*-Value
SFA						
C16:0	20.25	19.49	18.93	19.01	0.21	0.078
C18:0	9.73	9.12	8.86	8.74	0.17	0.189
C22:0	0.72 ^a^	0.61 ^b^	0.56 ^b^	0.57 ^b^	0.02	0.004
MUFA						
C16:1	1.27 ^c^	1.40 ^b^	1.38 ^b^	1.54 ^a^	0.02	<0.001
C18:1n-9c	22.25 ^c^	23.51 ^ab^	23.02 ^b^	24.04 ^a^	0.17	<0.001
C22:1n-9	5.80	5.07	6.16	6.48	0.26	0.265
C24:1	1.30 ^bc^	1.16 ^c^	1.55 ^ab^	1.68 ^a^	0.07	0.017
PUFA						
C18:2n-6	33.31 ^b^	35.38 ^a^	35.04 ^a^	35.53 ^a^	0.30	0.023
C18:3n-3	2.05 ^c^	2.02 ^c^	2.66 ^a^	2.32 ^b^	0.06	<0.001
C20:2	0.86 ^b^	0.75 ^b^	1.12 ^a^	1.22 ^a^	0.05	<0.001
C20:3n-6	0.34	0.33	0.35	0.37	0.01	0.58
C22:6n-3	0.60 ^c^	0.69 ^c^	1.21 ^a^	1.00 ^b^	0.06	<0.001
Total SFA	30.70 ^a^	29.21 ^ab^	28.35 ^b^	28.32 ^b^	0.34	0.037
Total MUFA	30.62 ^c^	31.13 ^bc^	32.10 ^b^	33.73 ^a^	0.33	0.001
Total PUFA	37.15 ^b^	39.15 ^a^	40.32 ^a^	40.43 ^a^	0.36	0.001
PUFA/SFA	1.22 ^b^	1.35 ^a^	1.43 ^a^	1.43 ^a^	0.03	0.004
n-6/n-3	57.83 ^a^	54.42 ^a^	29.82 ^b^	36.80 ^b^	2.99	<0.001

Note: The data were shown as means and standard error (SEM) (n = 6). The different lowercase letters in the same rows indicate significant difference (*p* < 0.05), while the same or without lowercase letters in the same rows indicate insignificant difference (*p* > 0.05). SFA: saturated fatty acid; MUFA: monounsaturated fatty acids; PUFA: polyunsaturated fatty acid; CON: basal diet group; ANT: supplement with 500 mg/kg oxytetracycline calcium; LAG: supplement with 150 mg/kg *Astragalus* polysaccharides and 75 mg/kg *Glycyrrhiza polysaccharides*; HAG: supplement with 300 mg/kg *Astragalus* polysaccharides and 150 mg/kg *Glycyrrhiza* polysaccharides.

**Table 5:** Effects of *Astragalus-Glycyrrhiza* polysaccharides on antioxidant function of broiler’s serum and breast muscle.

	Treatments					
Items	CON	ANT	LAG	HAG	SEM	*p*-Value
**Serum**						
T-AOC (U/mL)	8.15 ^b^	8. 37 ^b^	9.08 ^a^	8.89 ^a^	0.10	<0.001
GSH-Px (U/mL)	886.94 ^b^	889.72 ^b^	938.34 ^a^	906.35 ^b^	5.64	0.001
SOD (U/mL)	72.40 ^b^	75.85 ^b^	89.19 ^a^	79.47 ^b^	1.76	0.001
MDA (mmol/mL)	5.57 ^a^	5.62 ^a^	3.61 ^b^	3.65 ^b^	0.21	<0.001
**Breast muscle**						
T-AOC (U/mg prot)	127.98 ^b^	127.74 ^b^	135.54 ^a^	133.24 ^a^	1.13	0.002
GSH-Px (U/mg prot)	3.53	3.51	3.43	3.60	0.04	0.374
SOD (U/mg prot)	76.29	77.44	76.63	78.69	0.78	0.728
MDA (nmol/mg prot)	0.25 ^a^	0.24 ^a^	0.16 ^b^	0.16 ^b^	0.01	<0.001

Note: The data were shown as means and standard error (SEM) (n = 6). The different lowercase letters in the same rows indicate significant difference (*p* < 0.05), while the same or without lowercase letters in the same rows indicate insignificant difference (*p* > 0.05). T-AOC: total antioxidant capacity; GSH-Px: glutathione peroxidase; SOD: superoxide dismutase; MDA: malondialdehyde; CON: basal diet group; ANT: supplement with 500 mg/kg oxytetracycline calcium; LAG: supplement with 150 mg/kg *Astragalus* polysaccharides and 75 mg/kg *Glycyrrhiza polysaccharides*; HAG: supplement with 300 mg/kg *Astragalus* polysaccharides and 150 mg/kg *Glycyrrhiza* polysaccharides.

## Data Availability

The raw reads of sequencing data in this study have been uploaded to NCBI under the accession number of PRJNA793377.

## Supplementary Materials

The following supporting information can be downloaded at: https://www.mdpi.com/article/10.3390/antiox11101872/s1, Figure S1: (A) Shannon dilution curve analysis (B) Venn diagram analysis; Figure S2: (A) Orthogonal partial least squares discriminant analysis (OPLS-DA) and (C) response permutation testing (RPT) of serum metabolites between CON and LAG groups in positive ion mode. (B) Orthogonal partial least squares discriminant analysis (OPLS-DA) and (D) response permutation testing (RPT) of serum metabolites between CON and LAG groups in negative ion mode. R^2^X and R^2^Y represent the interpretation rate of the built model to the X and Y matrix, R^2^X (cum) and R^2^Y (cum) represent the cumulative interpretation rate; Q^2^ indicates the predictive power of the model.; Table S1: Composition and nutrient levels of basal diet (air-dry basis,%); Table S2: Primer sequences used for the quantitative real-time PCR.
