# Supplementary material for: Effects of Compound Polysaccharides Derived from Astragalus and Glycyrrhiza on Growth Performance, Meat Quality and Antioxidant Function of Broilers Based on Serum Metabolomics and Cecal Microbiota
Source: Antioxidants (Basel). 2022 Sep 21;11(10):1872. doi: 10.3390/antiox11101872 (PMC9598874; doi:10.3390/antiox11101872)
Supplement: Supplementary file 1 [file antioxidants-11-01872-s001.zip › antioxidants-1851833-supplementary.pdf]

Supplementary materials

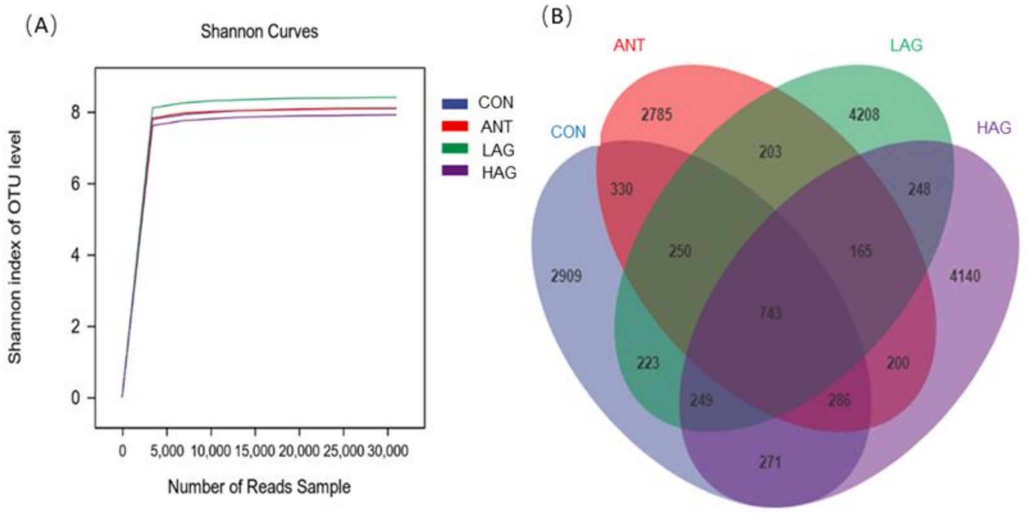

Figure S1. (A)Shannon dilution curve analysis (B)Venn diagram analysis

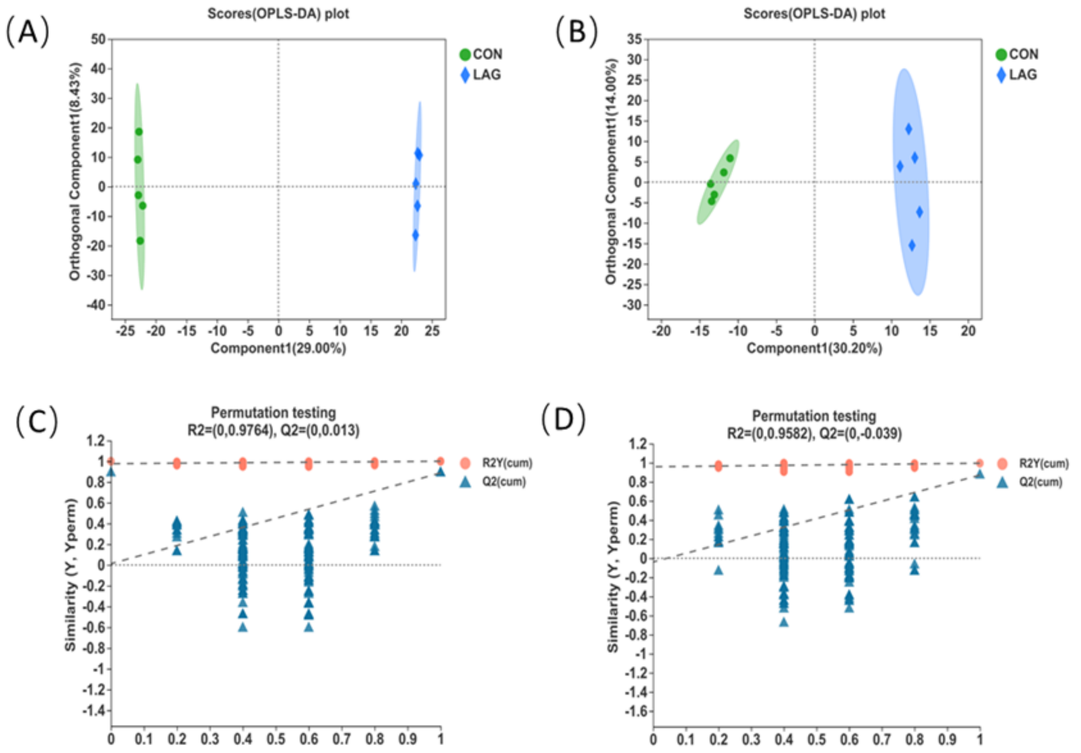

Figure S2. (A) Orthogonal partial least squares discriminant analysis (OPLS-DA) and (C) response permutation testing (RPT) of serum metabolites between CON and LAG groups in positive ion mode. (B) Orthogonal partial least squares discriminant analysis (OPLS-DA) and (D) response permutation testing (RPT) of serum metabolites between CON and LAG groups in negative ion mode. R^2X and R^2Y represent the interpretation rate of the built model to the X and Y matrix, R^2X (cum) and R^2Y (cum) represent the cumulative interpretation rate; Q^2 indicates the predictive power of the model.

Table S1. Composition and nutrient levels of basal diet (air-dry basis, %).

Items	1 to 21 days of age	22 to 42 days of age
Ingredients		
Corn	54.50	55.42
Soybean meal	29.70	25.30
Corn gluten meal	8.00	8.00
$CaHPO_4$	1.30	1.20
Limestone	1.40	1.40
NaCl	0.30	0.30
Soybean oil	2.80	6.50
Soda	0.15	0.15
L-Lysine	0.87	0.80
DL-Methionine	0.25	0.21
Threonine	0.13	0.12
Premix[1]	0.60	0.60
Total	100.00	100.00
Nutrient levels[2]		
Metabolizable energy (MJ/kg)	12.61	13.59
Crude protein	23.39	21.19
Calcium	0.77	0.72
Total phosphorus	0.56	0.54
Lysine	1.50	1.34
Methionine	0.62	0.55
Threonine	0.97	0.88

[1] Supplied per kilogram of diet: for 1 to 21d, 12000 IU vitamin A; 4500 IU vitamin D_3; 30 IU vitamin E; 4.5 mg vitamin K; 2.8 mg vitamin B_1; 9.6 mg vitamin B_2; 3.75 mg vitamin B_6; 30μg vitamin B_{12}; 49.5 mg niacin; 20 mg calcium pantothenate; 1.5 mg folic acid; 0.18 mg biotin; 500 mg choline; 100 mg Zn; 110 mg Fe; 20 mg Cu; 120 mg Mn; 0.7mg I; 0.3 mg Se. For 22 to 42d, 10000 IU vitamin A; 3750 IU vitamin D_3; 25 IU vitamin E; 3.75 mg vitamin K; 2.3 mg vitamin B_1; 8 mg vitamin B_2; VB_6，3.1 mg vitamin B_6; 25 μg vitamin B_{12}; 41.2 mg niacin; 20 mg calcium pantothenate; 1.25 mg folic acid; 0.12 mg biotin; 400 mg choline; 100 mg Zn; 110 mg Fe; 20 mg Cu; 120 mg Mn; 0.7mg I; 0.3 mg Se. [2] Metabolizable energy was calculated, while the others were measured.

Table S2. Primer sequences used for the quantitative real-time PCR.

Gene	Sequence (5’–3’)	Accession number	Amplicon size (bp)
SOD1	Forward: AAATGGGTGTACCAGCGCA<br>Reverse: CTTTGCAGTCACATTGCCGA	NM_205064.1	109
SOD2	Forward: GAGGGGAGCCTAAAGGAGAAT<br>Reverse: TCTTGATTTGCACAGGCTGC	NM_204211.1	175
GSH-PX1	Forward: TCACCATGTTCGAGAAGTGC<br>Reverse: ATGTACTGCGGGTTGGTCAT	NM_001277853.1	124
GAPDH	Forward: AGCCATTCCTCCACCTTTGAT<br>Reverse: AGTCCACAACACGGTTGCTGTAT	NM_204305	112

Note: *SOD1*: superoxide dismutase 1; *SOD2*: superoxide dismutase 2; *GSH-Px*: glutathione peroxidase; *GAPDH*: glyceraldehyde 3-phosphate dehydrogenase.
